# Improved sensitivity of an interferon-gamma release assay (T-SPOT.TB™) in combination with tuberculin skin test for the diagnosis of latent tuberculosis in the presence of HIV co-Infection

**DOI:** 10.1186/1471-2334-11-319

**Published:** 2011-11-15

**Authors:** Luigia Elzi, Ingrid Steffen, Hansjakob Furrer, Jan Fehr, Matthias Cavassini, Bernard Hirschel, Matthias Hoffmann, Enos Bernasconi, Stefano Bassetti, Manuel Battegay

**Affiliations:** 1 Division of Infectious Diseases and Hospital Epidemiology, University Hospital Basel, Basel, Switzerland; 2 Institute of Medical Microbiology, University of Basel, Basel, Switzerland; 3 University Clinic for Infectious Diseases, University Hospital Bern and University of Bern, Bern, Switzerland; 4 Division of Infectious Diseases and Hospital Epidemiology, University Hospital Zurich, Zurich, Switzerland; 5 Division of Infectious Diseases, University Hospital Lausanne, Lausanne, Switzerland; 6 Division of Infectious Diseases, University Hospital Geneva, Geneva, Switzerland; 7 Cantonal Hospital, St. Gallen, Switzerland; 8 Regional Hospital, Lugano, Switzerland; 9 Cantonal Hospital, Olten, Switzerland

## Abstract

**Background:**

Interferon-gamma release assays (IGRA) are more specific than the tuberculin skin test (TST) for the diagnosis of *Mycobacterium tuberculosis *infection. Data on sensitivity are controversial in HIV infection.

**Methods:**

IGRA (T-SPOT.TB) was performed using lymphocytes stored within 6 months before culture-confirmed tuberculosis was diagnosed in HIV-infected individuals in the Swiss HIV Cohort Study.

**Results:**

64 individuals (69% males, 45% of non-white ethnicity, median age 35 years (interquartile range [IQR] 31-42), 28% with prior AIDS) were analysed. Median CD4 cell count was 223 cells/μl (IQR 103-339), HIV-RNA was 4.7 log_10 _copies/mL (IQR 4.3-5.2). T-SPOT.TB resulted positive in 25 patients (39%), negative in 18 (28%) and indeterminate in 21 (33%), corresponding to a sensitivity of 39% (95% CI 27-51%) if all test results were considered, and 58% (95% CI 43-74%) if indeterminate results were excluded. Sensitivity of IGRA was independent of CD4 cell count (p = 0.698). Among 44 individuals with available TST, 22 (50%) had a positive TST. Agreement between TST and IGRA was 57% (kappa = 0.14, p = 0.177), and in 34% (10/29) both tests were positive. Combining TST and IGRA (at least one test positive) resulted in an improved sensitivity of 67% (95% CI 52-81%). In multivariate analysis, older age was associated with negative results of TST and T-SPOT.TB (OR 3.07, 95% CI 1,22-7.74, p = 0.017, per 10 years older).

**Conclusions:**

T-SPOT.TB and TST have similar sensitivity to detect latent TB in HIV-infected individuals. Combining TST and IGRA may help clinicians to better select HIV-infected individuals with latent tuberculosis who qualify for preventive treatment.

## Background

Tuberculosis (TB) continues to be a global public health epidemic with 2 million deaths yearly [1]. One third of the world's population is latently infected with *Mycobacterium tuberculosis *[2]. HIV-infected individuals are particularly susceptible to TB, both from new infection with rapid progression to active disease and reactivation of latent infection occurring in 20-30% of subjects with a positive tuberculin skin test (TST) [3-5].

There is still no reliable test to detect latent TB. The TST with purified protein derivative (PPD) by the Mantoux method has a limited sensitivity especially in HIV-infected individuals [6-8], even after the introduction of combination antiretroviral therapy [9]. Limitations of TST include reader variability, false-positive results due to cross-reactivity with environmental mycobacteria and previous Bacillus Calmette-Guérin (BCG) vaccination, and false-negative results due to anergy in immunosuppressed individuals [7]. Recently, interferon-gamma based assays (IGRA) to detect specific cellular immune response to antigens expressed in *M. tuberculosis *(ESAT-6: early secretory antigenic target 6, and CFP-10: culture filtrate protein 10), but absent in BCG and many environmental mycobacteria, have been reported to improve sensitivity and specificity for the diagnosis of TB [10-12]. Regarding sensitivity, however, published studies in this area are highly variable in respect to sample size, type of assay, interpretation criteria, study population and TB endemic setting. Since IGRA rely on immune response, their performance may be impaired in HIV-infection [11,13-15].

IGRA are already part of clinical practice in several countries with low prevalence of TB [16]. In Switzerland, 2 IGRA are currently commercially available: a whole-blood (QuantiFERON-TB Gold In-Tube™, Cellestis Ltd. Victoria, Australia) and an enzyme-linked immunospot assay (T-SPOT.TB™, Oxford Immunotec Ltd., Abingdon, UK) [17]. Previous studies suggested that sensitivity of T-SPOT.TB is less impaired than QuantiFERON in the setting of advanced immunosuppression and HIV-infection [10,18,19].

We aimed to evaluate the sensitivity of T-SPOT.TB in comparison to TST to identify HIV-infected individuals with latent TB, who therefore qualify for preventive treatment. All included patients developed culture-confirmed TB within 6 months; from this we deduce that they had latent TB when they were enrolled in the Swiss HIV Cohort Study and they were sampled.

## Methods

### Study design

T-SPOT.TB was retrospectively performed using frozen viable lymphocytes of HIV-infected individuals participating in the Swiss HIV Cohort Study (SHCS) that had been stored within 6 months before culture-confirmed TB occurred. Performance of T-SPOT.TB was compared with TST, when available.

### Study population

The SHCS [20] is a large prospective cohort study with continuous enrolment of adult HIV-infected individuals. Basic socio-demographic characteristics, data on the clinical course (occurrence of opportunistic infections, death), co-infection with hepatitis B and C, TST, antiretroviral therapy, co-medication (prophylaxis and treatment of opportunistic infections), immunologic and virologic parameters are collected at enrolment into the study and every 6 months thereafter on standardised data collection forms. At registration and at every follow-up visit, plasma samples are frozen for further analysis. In addition, once a year a sample of viable peripheral blood mononuclear cells (PBMC) is stored (three aliquots of at least 1.5 million cells). AIDS-defining diseases are recorded using the 1993 revised clinical definition of AIDS from the Centers for Disease Control and Prevention [21]. The cause of death is reported using the 10^th ^revision of the International Classification of Diseases and Related Health Problems (ICD-10) [22].

### Laboratory assay

T-SPOT.TB™ (Oxford Immunotec Ltd., Abingdon, UK) is a simplified variant of the enzyme-linked immunospot (ELISPOT) assay technique for the determination of effector T-cells which secrete interferon gamma in response to stimulation by antigens specific for *M. tuberculosis*. T-SPOT.TB was performed by using a commercial kit according to the manufacturer's instructions [17]. Each patient test required 4 wells: one for the negative control (containing no antigen), one for the positive control (phytohaemagglutinin) and 2 for the *M. tuberculosis *antigens, Panel A (ESAT-6) and B (CFP-10). Evaluating the number of spots obtained provided a measurement of the frequency of *M. tuberculosis *sensitive cells. The test result was considered "positive" if the number of spots per test well was ≥ 6 in either of both Panel A and B. The test result was considered "negative" if both Panel A and B showed < 6 spots. Where the positive control was < 20 spots, or the negative control ≥ 10 spots, the test was scored as "indeterminate". Of note, interpretation criteria of T-SPOT.TB by Food and Drug Administration (FDA) for the use of the test in the USA differ from those used in other countries, where an "indeterminate" test result occurs if the negative control is either > 10 spots, or ≤ 10 spots but the positive control is < 20 spots and Panel A or B < 5 spots. According to USA guidelines, a borderline test result occurs if Panel A or B is between 5 and 7 spots and the negative control ≤ 10 spots [19].

### Viability of stored frozen lymphocytes

We assumed that T-SPOT.TB was feasible using stored frozen lymphocytes. According to Oxford Immunotec Ltd., freezing of PBMC is possible, but the use of fresh white blood cells is recommended because of the risk of some deterioration in the performance of the assay [Oxford Immunotec, Technical bulletins Nr 1, 4, 10, 2005].

Following steps were undertaken to guarantee the good performance and the correct interpretation of the test using frozen lymphocytes: a) Time between collection and freezing of samples, and procedures of freezing and storing PBMC were performed according to a specific protocol of the SHCS, assuring comparable quality of stored cells in all study centers. Viability of frozen PBMC was checked regularly. b) After thawing, viable cells were counted using the trypan blue dye exclusion method, confirming that stored cells were still able to produce interferon gamma. Recovery had to be more than 70% of the original input. The procedures a) and b) were validated in several studies performed within the framework of the Swiss HIV Cohort Study [23-25]. c) For each patient, a negative control (without antigen) and a positive control (proliferation assay) were performed, the last one with addition of phytohaemagglutinin mitogens, which induce the production of gamma interferon, confirming again the viability of the cells used for the IGRA. The 2 wells containing *M. tuberculosis *specific antigens (ESAT-6 and CFB-10) were only read if both negative and positive controls had been performed and were valid according to the manufacturer's protocol.

### Statistical analysis

Basic socio-demographic characteristics, CD4 cell count, HIV viral load, and antiretroviral therapy were compared using the Chi-square test or Fisher's exact test for categorical variables, and the Mann-Whitney or Kruskal-Wallis test for continuous variables. Logistic regression was used to assess factors associated with a positive T-SPOT.TB result, and with negative results of both T-SPOT.TB and TST. We built the final model using a forward stepwise approach, adding each factor significant at the level of 0.1 in the model one by one. Agreement between T-SPOT.TB and TST was determined by calculation of Cohen's kappa coefficient.

All analyses were performed using STATA™ software version 11 for Windows (STATA Corp, College Station, Texas, USA).

### Ethical approval

The SHCS was approved by the relevant ethics committees of the participating centres, and written consent was obtained from all study participants.

## Results

### Study population

Among 242 HIV-infected individuals who developed culture-confirmed TB between 1993 and 2005 after enrolment in the SHCS, we identified 64 subjects with a sample of viable lymphocytes collected prior to TB diagnosis that could be analyzed. Table 1 summarises the baseline characteristics of participants. Most patients (69%) were males, the median age was 35 years (interquartile range [IQR] 31-42), 45% were of non-white ethnicity, and 18% had previously been diagnosed with an AIDS defining condition. The median CD4 cell count was 223 cells/μL (IQR 103-339), and the median HIV-RNA was 4.7 copies/mL (IQR 4.3-5.2) at the time of cells sampling prior to TB diagnosis. Culture-confirmed TB was diagnosed after a median of 680 days (IQR 208-1948) after enrolment in the SHCS.

**Table 1:** General characteristics of the study population (n = 64) according to results of T-SPOT.TB assay

Variable		Positive T-SPOT.TB		Negative T-SPOT.TB		Indeterminate T-SPOT.TB		p-value
		N = 25		N = 18		N = 21		
		n	%	n	%	n	%	
Median age, IQR (years)		35	32-40	36	30-43	35	29-44	0.915
Males		18	72	12	67	14	67	0.904
Median body mass index, IQR (kg/m^2^)		22	20-25	22	21-25	23	20-26	0.906
White ethnicity		13	52	7	39	9	53	0.670
Prior AIDS-defining condition		5	20	5	25	8	38	0.396
Risk	MSM	5	20	4	22	7	28	0.978
	Heterosexual	13	52	10	56	4	22	
	Intravenous drug use	7	38	10	48	5	24	
TB disease	Pulmonary	13	52	7	39	13	62	0.357
	Extrapulmonary	8	32	9	50	7	33	
	Pulmonary and extrapulmonary	4	16	2	11	1	5	
Median CD4 cell count at TB diagnosis, IQR (cells/μL)		235	106-330	169	70-297	229	130-300	0.572
Median HIV-RNA at TB diagnosis, IQR (log_10 _copies/mL)		4.7	3.5-5.6	5.0	4.6-5.2	4.9	4.6-5.3	0.531
Median CD4 cell count at cells sampling, IQR (cells/μL)		235	122-320	147	70-349	256	108-379	0.498
Median CD8 cell count at cells sampling, IQR (cells/μL)		614	448-889	842	460-1191	835	510-1417	0.308
Median HIV-RNA at cells sampling, IQR (log_10 _copies/mL)		4.8	3.2-5.3	4.9	4.6-5.0	4.6	4.3-5.1	0.868
Antiretroviral therapy at cells sampling		13	52	14	78	12	57	0.211
Time between cells sampling and TB diagnosis, IQR (days)		63	15-126	57	12-163	72	20-284	0.786
Time between cells sampling and performance of IGRA, IQR (months)		81	48-135	107	70-141	123	84-167	0.142
TST	≥ 5 mm	10	40	7	39	5	24	0.414
	< 5 mm	7	28	8	44	7	33	
	missing	8	32	3	17	9	43	

### T-SPOT.TB

The IGRA assay was performed using lymphocytes obtained at a median of 91 days (IQR 47-167) before active tuberculosis was diagnosed. T-SPOT.TB assay resulted positive in 25 patients (39%), negative in 18 (28%) and indeterminate in 21 (33%), corresponding to a sensitivity of 39% (95% confidence interval [CI] 27-51%) if all test results were considered, and 58% (95% CI 43-74%) if indeterminate results were excluded. Among 21 indeterminate test results, 9 (43%) had a positive nil control and 12 (57%) a fair mitogen control, i.e. there were less than 20 positive spots.

**Figure 1:**
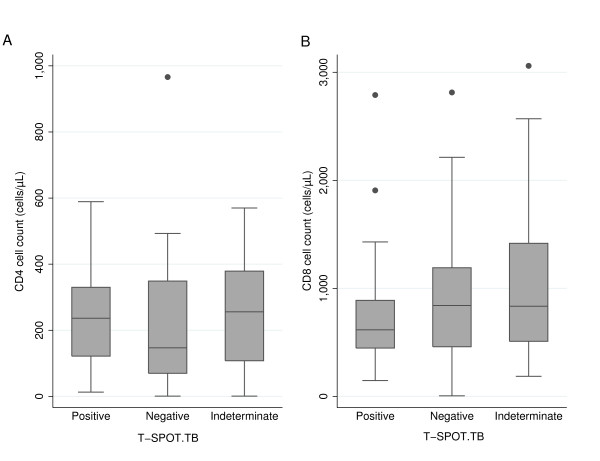
**Results of T-SPOT.TB assay according to (A) CD4 cell count and (B) CD8 cell count**.

Overall, there was no significant correlation between results of T-SPOT.TB and the degree of immunosuppression as measured by the CD4 cell count (Spearman correlation coefficient r = 0.02) (Figure 1). However, in patients with a positive T-SPOT.TB result we observed an association between higher number of spots and increasing CD4 cell count (p = 0.037), but not with CD8 cell count (p = 0.244), suggesting improved sensitivity of T-SPOT.TB in subjects with less advanced immunosuppression. In both univariate and multivariate analyses, regardless whether indeterminate results were excluded from the analysis, sensitivity of T-SPOT.TB was independent of age, sex, body mass index, ethnicity, HIV viral load, HIV clinical stage, antiretroviral treatment, and time between freezing of cells samples and performance of IGRA (Table 2).

**Table 2:** Factors associated with a positive T-SPOT.TB in 43 patients with determinate test results. Univariate and multivariate analysis

Variable		Univariate analysis			Multivariate analysis		
		OR	95% CI	p-value	Adjusted OR	95% CI	p-value
Age, per 10 years older		1.02	0.52-2.03	0.945	1.23	0.53-2.87	0.624
Female		0.78	0.21-2.89	0.707	1.36	0.17-11.1	0.775
Body mass index, per 5 kg/m^2 ^increase		0.62	0.20-1.89	0.403	0.59	0.18-2.00	0.398
Non-white ethnicity		1.70	0.50-5.83	0.397	-	-	-
Risk	Men who have sex with men	1*	-	-	-	-	-
	Heterosexual	1.04	0.22-4.91	0.960	-	-	-
	Intravenous drug use	1.40	0.23-8.46	0.714	-	-	-
Prior AIDS-defining condition		0.65	0.16-2.70	0.553	-	-	-
Antiretroviral treatment		0.31	0.26-1.21	0.120	0.87	0.14-5.65	0.888
CD4 cell count, per 50 cells/μL increase		1.01	0.85-1.20	0.904	-	-	-
CD4 cell count ≥200 versus < 200 cells/μL		2.55	0.72-8.96	0.146	1.41	0.27-7.39	0.682
CD8 cell count, per 50 cells/μL increase		0.97	0.93-1.02	0.292	-	-	-
Positive TST (≥ 5 mm)		1.63	0.40-6.63	0.493	-	-	-
Extrapulmonary versus pulmonary TB		2.13	0.61-7.41	0.237	-	-	-
Time between cells freezing and IGRA performance, per month		0.99	0.98-1.01	0.453	-	-	-

* Reference category

The probability of scoring an indeterminate T-SPOT.TB result in our study was not related to the degree of immunosuppression, antiretroviral treatment or time between lymphocytes sampling and performance of IGRA.

### Tuberculin skin test

Among 44 individuals with available TST results, 22 had a positive TST, defined as a skin induration of ≥ 5 mm, corresponding to a sensitivity of 50% (95% CI 35-65%). The median time between performance of TST and diagnosis of active TB was 379 days (IQR 70-1267).

**Figure 2:**
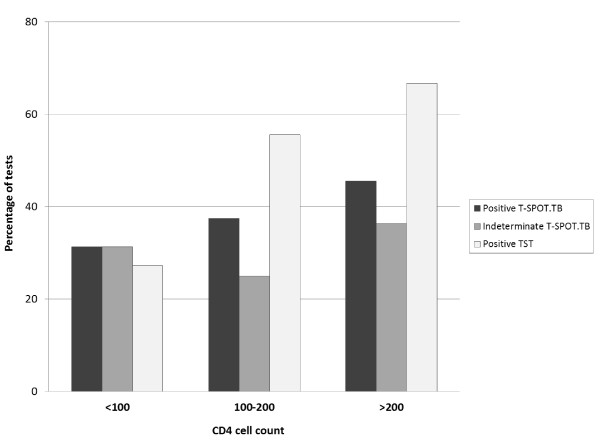
**Proportion of positive test results of TST and T-SPOT.TB and indeterminate T-SPOT.TB results according to CD4 cell count (test for trend p = 0.135 for the association between CD4 cell count and T-SPOT.TB)**.

In contrast to T-SPOT.TB, sensitivity of TST was clearly dependent on age and CD4 cell count, being lower in older patients and in those with more advanced immunosuppression (Figure 2). In multivariate analysis, after adjustment for age, gender, ethnicity, HIV clinical stage, CD4 cell count and antiretroviral therapy, a positive TST test result was associated with age (OR 0.17, 95% CI 0.05-0.63, p = 0.008, per 10 years older) and CD4 cell count (OR 5.11, 95% CI 1.04-26.4, p = 0.022 for CD4 cell count above 100 cells/μl).

### Comparison between T-SPOT.TB and TST

Among 44 patients with available results of TST and T-SPOT.TB, overall agreement between both tests was noted in 57% of patients (kappa = 0.14, p = 0.177). In 29 subjects with positive test results by either TST or T-SPOT.TB, only 10 (34%) had positive results with both modalities.

Among 22 individuals with a positive TST, 10 (45%) had also a positive T-SPOT.TB, 7 (32%) a negative and 5 (23%) an indeterminate IGRA result, whereas among 22 individuals with a negative TST, 7 (32%) had a positive T-SPOT.TB, 8 (36%) a negative, and 7 (32%) an indeterminate IGRA result (p = 0.745). If both TST and T-SPOT.TB results were combined (at least one test positive) and indeterminate results excluded a sensitivity of 67% (95% CI 52-81%) was reached.

In univariate and multivariate analyses (Table 3), older age was the only risk factor of having negative test results with both TST and T-SPOT.TB (OR 3.07, 95% CI 1,22-7.74, p = 0.017, per 10 years older).

**Table 3:** Factors associated with negative test results of both T-SPOT.TB and TST

Variable		Univariate analysis			Multivariate analysis		
		OR	95% CI	p-value	Adjusted OR	95% CI	p-value
Age, per 10 years older		2.93	1.24-6.91	0.014	3.07	1.22-7.74	0.017
Female		0.96	0.20-4.52	0.073	2.22	0.33-14.7	0.409
Body mass index, per 5 kg/m^2 ^increase		1.71	0.62-4.69	0.300			
Non-white ethnicity		0.22	0.04-1.55	0.092	-	-	-
Risk	Men who have sex with men	1*	-	-	-	-	-
	Heterosexual	0.25	0.15-1.14	0.134	-	-	-
	Intravenous drug use	0.67	0.13-3.45	0.629	-	-	-
Prior AIDS-defining condition		3.35	0.87-13.0	0.080	3.02	0.56-16.2	0.198
Antiretroviral treatment		1.24	0.31-4.96	0.764	0.71	0.13-4.01	0.699
CD4 cell count, per 50 cells/μL increase		0.86	0.70-1.04	0.123	-	-	-
CD4 cell count ≥200 versus < 200 cells/μL		0.41	0.11-1.46	0.168	0.46	0.10-2.05	0.306
CD8 cell count, per 50 cells/μL increase		1.01	0.96-1.05	0.740	-	-	-
Extrapulmonary versus pulmonary TB		1.22	0.34-4.32	0.759	-	-	-

## Discussion

This study evaluating an interferon-gamma release assay, T-SPOT.TB, in 64 HIV-infected adults who all developed culture-confirmed TB after enrolment in the Swiss HIV Cohort Study indicates that T-SPOT.TB has a similar sensitivity to TST to detect latent TB in HIV-infected individuals. However, there was poor agreement between T-SPOT.TB and TST results. In contrast to TST, sensitivity of T-SPOT.TB was independent of the level of immunodeficiency, although a trend towards higher number of spot forming units with increasing CD4 cell count was observed in patients with a positive T-SPOT.TB. Importantly, combination of TST and T-SPOT.TB with at least one test positive resulted in improved sensitivity. This is likely to be useful in clinical practice to better identify HIV-infected individuals with latent TB who qualify for preventive treatment.

Sensitivity of T-SPOT.TB of 39-58% in our study was lower than in published studies [10,11,23,26-28]. In the absence of a gold standard to diagnose latent or culture negative TB, assessments of accuracy of tests for TB are difficult, especially in the context of immunodeficiency. So far, sensitivity of IGRA for latent TB has been mainly estimated by comparison with TST in cross-sectional studies, or by assessing the number of positive IGRA results in patients with culture-confirmed TB. Because TST and IGRA are indirect tests that measure immunologic responses and do not detect the causative organism, assessment of sensitivity among persons with positive TST or active TB might not reliably estimate sensitivity for latent TB. In our study, T-SPOT.TB was performed in a highly selected population, namely HIV-infected individuals shortly before they developed culture-confirmed TB, providing a reliable approximation of patients with latent TB in an area of low TB transmission, since reinfection with *M. tuberculosis *was unlikely. The quite low sensitivity of T-SPOT.TB in our population was independent of socio-demographic characteristics, including ethnicity and country of origin, level of immunodeficiency and antiretroviral treatment, suggesting that immunologic differences implied in the progression of latent infection to active disease might negatively affect IGRA results. The suboptimal sensitivity of Interferon-based in-vitro assays, especially in immunocompromised individuals, emphasizes the need for alternative markers for diagnosing tuberculosis, such as interferon-inducible protein (IP-10), which appeared less influenced by HIV infection. Further studies are needed to test the clinical impact of these findings [15,29].

The major concern in using T-cell based assays in the setting of HIV infection is the influence of immunodeficiency on sensitivity. While some studies suggested that IGRA were less influenced by HIV infection [15,18,26-28,30,31] other studies reported loss of sensitivity with severe immunodeficiency, in particular when CD4 cell counts were less than 100 cells/μl [15,28,30,32-35]. We observed a clear association between CD4 cell count and TST but not between CD4 cell count and T-SPOT.TB, although a trend towards higher number of spot forming cells with increasing CD4 cell count was noted in patients with positive T-SPOT.TB result. As both tests rely on a T-cell mediated immune response, this difference might also result from the cut-off chosen for scoring a positive T-SPOT.TB. A recent publication suggests that accuracy in HIV-infected patients improves when the number of spot forming cells from the T-SPOT.TB test is related to CD4 count [36].

In line with prior studies [18,28,37-39], we observed poor agreement between TST and T-SPOT.TB. However, combination of TST and T-SPOT.TB with at least one test positive was shown to enhance sensitivity to detect latent TB in HIV co-infection. This is clinically relevant, since easier and more accurate diagnosis of latent TB implies start of preventive treatment, which was demonstrated to be highly effective [9]. In multivariate analysis, older age was the only risk factor of scoring both tests negative, indicating a limitation of this strategy.

The high number of indeterminate test results in our study was not associated with lower CD4 cell counts in contrast to other reports [37,38,40]. As no correlation between time of lymphocyte sampling and performance of T-SPOT.TB was noted, it is also unlikely that the quality of cell samples was impaired.

### Limitations and strengths

We acknowledge some limitations. The use of frozen stored lymphocytes to perform T-SPOT.TB might have impaired the ability of producing gamma-interferon, leading to an underestimation of sensitivity. However, viability of cells was meticulously checked, validated in several studies of the Swiss HIV Cohort Study [23-25], and T-SPOT.TB was always performed together with mitogens and nil controls, as described in the method section. As TB negative subjects were not included in our study, we could not estimate specificity of T-SPOT.TB and TST. However, the focus of this study was on sensitivity, because improvement of sensitivity is the main diagnostic need in latent TB in HIV-infected individuals. Due to the retrospective character of this study, results of TST were not available for all patients, and information on previous BCG vaccination was not collected in the SHCS database. Due to the small amount of viable peripheral blood mononuclear cells routinely stored in the Swiss HIV Cohort Study (3 aliquots of 1.5 million cells) we were not able to perform additional analysis such as the measurement of T regulatory cells. Moreover, we were not able to repeat T-SPOT.TB at later time points before culture-confirmed active tuberculosis was diagnosed, because cells samples were collected only once a year after enrolment in the Swiss HIV Cohort Study according to the protocol.

Strengths of this study were the inclusion of HIV-infected individuals with latent TB who all developed culture-confirmed TB in a low endemic area, and evaluation of combined testing with TST to enhance sensitivity of T-SPOT.TB. This is clinically relevant, since easier and more accurate diagnosis of latent TB implies start of preventive chemotherapy, that was shown to be highly effective in HIV-infected individuals [9].

## Conclusion

In conclusion, T-SPOT.TB and TST have similar sensitivity to detect latent TB in HIV-infected individuals. Combination of TST and T-SPOT.TB with at least one test positive resulted in enhanced sensitivity. This is likely to help clinicians in their decision to start treatment of latent TB in HIV-infected individuals. Further studies are needed to confirm our findings and to evaluate newer biomarkers like IP-10 for the diagnosis of tuberculosis in the setting of immunodeficiency and in particular HIV infection.

## Competing interests

H. Furrer has received grants from GlaxoSmithKline, Bristol-Myers Squibb, Gilead, Merck, Boehringer-Ingelheim. J. Fehr has received grants and speakers' honoraria from Merck, Gilead, Janssen. M. Cavassini has received travel grants from Abbott, Gilead, Roche, Boehringer Ingelheim. B. Hirschel has received travel grants and speakers' honoraria from Abbott, Bristol-Myers Squibb, Gilead, GlaxoSmithKline, Merck, Roche. M. Hoffmann has received travel grants and/or speakers' honoraria from Cellestis, Roche, Bristol-Myers Squibb, Janssen. E. Bernasconi has received travel grants or honoraria from Gilead, Roche, GlaxoSmithKline, Pfizer, Boehringer Ingelheim, Janssen. M. Battegay has received research grants or speakers' honoraria from Abbott, Bristol-Myers Squibb, Boehringer Ingelheim, GlaxoSmithKline, Roche, Merck, TRB Chemedica, and Janssen.

## Authors' contributions

All authors have read and approved the present manuscript.

LE, HF, SB, and MB have developed the research question, designed the study, collected data, interpreted results and written the manuscript. LE has performed the statistical analysis. IS has performed interferon-gamma release assay and interpreted results. JF, BH, MC, MH, and EB have collected data and interpreted results.

## Pre-publication history

The pre-publication history for this paper can be accessed here:

http://www.biomedcentral.com/1471-2334/11/319/prepub
